# Functional Near-Infrared Spectroscopy in Hearing Loss: A Systematic Review of Cortical Responses in Distinct Clinical Populations

**DOI:** 10.3390/brainsci16050532

**Published:** 2026-05-18

**Authors:** Valeria Del Vecchio, Giovanni Freda, Andrea de Bartolomeis, Nicola Serra, Domenico D’Errico, Salvatore Allosso, Elena Cantone, Davide Brotto, Judit Gervain, Patrizia Trevisi, Anna Rita Fetoni

**Affiliations:** 1 Hearing and Balance Unit, Department of Head and Neck, Federico II University Hospital, 81031 Naples, Italy; valeria.delvecchio@unina.it (V.D.V.); dome.derrico1989@gmail.com (D.D.); annarita.fetoni@unina.it (A.R.F.); 2 Audiology Section, Department of Neuroscience and Reproductive Sciences and Dentistry, University of Naples Federico II, 81031 Naples, Italy; nicola.serra@unina.it; 3 Section of Psychiatry, Laboratory of Translational and Molecular Psychiatry, Unit of Treatment-Resistant Psychosis, Department of Neuroscience and Reproductive Sciences and Dentistry, University of Naples Federico II, 81031 Naples, Italy; adebarto@unina.it; 4 Psychiatry and Psychology Unit, Department of Head and Neck, Federico II University Hospital, 81031 Naples, Italy; 5 Otorhinolaryngology Unit, Department of Head and Neck, Federico II University Hospital, 81031 Naples, Italy; salvatore.allosso@gmail.com; 6 Department of Pharmacy and Health and Nutrition Sciences, ENT Section, University of Calabria, 87036 Rende, Italy; elena.cantone@unical.it; 7 Department of Developmental and Social Psychology, University of Padua, 35128 Padua, Italy; davide.brotto@unipd.it (D.B.); judit.gervain@unipd.it (J.G.); 8 Section of Otolaryngology, Department of Neuroscience DNS, University of Padua, 35128 Padua, Italy; patrizia.trevisi@unipd.it; 9 Padova Neuroscience Center, University of Padua, 35128 Padua, Italy; 10 Integrative Neuroscience and Cognition Center, Université Paris Cité & CNRS, 75006 Paris, France

**Keywords:** functional near-infrared spectroscopy, auditory–cognitive load, cortical plasticity, cochlear implant

## Abstract

**Highlights:**

**What are the main findings?**

- Functional near-infrared spectroscopy (fNIRS) studies in adults with hearing loss reveal task-dependent and population-specific cortical activation patterns, rather than a unified or comparable neuroplastic response across conditions.
- In age-related hearing loss (ARHL), increased prefrontal recruitment is consistently associated with listening effort and processing inefficiency, rather than effective compensation.
- In cochlear implant (CI) users, cortical activation reflects language-related processing and audiovisual sensitivity within a distinct neurobiological and rehabilitative context, not directly comparable to ARHL.

**What are the implications of the main findings?**

- The available evidence supports fNIRS as a promising tool for investigating cortical responses to auditory and cognitive task demands within specific populations, but does not support cross-population generalization or unified models of plasticity across the lifespan.
- The present review is therefore descriptive and population-stratified rather than mechanistically integrative, and should not be interpreted as evidence of shared underlying mechanisms or as supporting a common explanatory framework between ARHL and CI users.
- Future research should prioritize standardized methodologies and longitudinal designs to clarify within-population mechanisms and their clinical relevance.

**Abstract:**

**Background/Objectives**: Functional near-infrared spectroscopy (fNIRS) has emerged as a non-invasive, implant-compatible imaging modality capable of capturing cortical hemodynamics during ecologically valid auditory and linguistic tasks. Its silent operation and tolerance to electrical artifacts make it particularly well suited to the study of hearing-impaired individuals, including cochlear implant (CI) users. However, evidence on the application of fNIRS to investigate speech perception, cognitive performance, and proxy of cortical activation patterns in patients with hearing loss (HL) remains fragmented. This systematic review aims to provide a structured, population-stratified description of current fNIRS literature on auditory and cognitive processing in adults with age-related hearing loss (ARHL) and CI users. **Methods**: A systematic search on PubMed Central, Web of Science and Scopus, based on PRISMA (2020) guidelines, was conducted to identify original studies that evaluate speech perception by means of fNIRS to assess auditory and cognitive process in hearing-impaired populations. **Results**: Across studies, fNIRS consistently detected activation of superior temporal and frontal cortices during speech-related tasks. In ARHL, increased dorsolateral prefrontal cortex (DLPFC) recruitment during speech-in-noise indicated compensatory yet inefficient processing. Longitudinal auditory training led to reduced prefrontal overactivation and enhanced temporal–frontal connectivity. In CI users, cortical responses to phonological and comprehension tasks show partially overlapping activation patterns with normal hearing (NH) peers, although arising within different neurobiological contexts, and are modulated by device experience and residual hearing (AV) speech, and stimulus-level effects further shape cortical responses. When interpreted in light of developmental evidence, these findings may be contextualized as reflecting distinct trajectories of cortical reorganization, rather than a common mechanism. **Conclusions**: fNIRS provides a tool to investigate auditory and cognitive responses in distinct hearing-impaired populations under ecologically valid conditions. It detects maladaptive frontal inefficiency in ARHL, tracks neuroplastic changes after rehabilitation, and captures population-specific cortical recruitment patterns in CI users. These findings are descriptive and context-dependent, and do not support cross-population mechanistic generalizations. Standardized protocols and longitudinal pediatric studies are needed to clarify the potential clinical relevance of fNIRS-derived cortical measures.

## 1. Introduction

Functional near-infrared spectroscopy (fNIRS) has become a very promising tool for studying the human auditory system in vivo, especially in conditions where conventional neuroimaging cannot be implemented easily. Unlike functional magnetic resonance imaging (fMRI) or positron emission tomography, fNIRS is silent, portable, and compatible with hearing devices such as cochlear implant (CI), avoiding artifacts related to strong magnetic fields or electrical stimulation [1]. These characteristics make it particularly suitable for investigating naturalistic speech processing and listening effort in real-world scenarios, an essential feature for patients with hearing loss (HL) who often struggle in complex acoustic environments. By monitoring task-evoked changes by means of oxygenated and deoxygenated hemoglobin, fNIRS provides a direct window on cortical hemodynamics in key auditory and cognitive regions during listening, speech perception, and multimodal integration tasks [2]. Its safety and feasibility in populations traditionally excluded from fMRI—including older adults with age-related hearing loss (ARHL) and individuals with CI—have contributed to its rapid diffusion in auditory neuroscience. Importantly, although fNIRS requires substantially lower infrastructure and operational costs than MRI-based techniques, it remains more expensive than electrophysiological systems such as electroencephalography (EEG) or auditory evoked potentials. Therefore, fNIRS is more appropriately considered an intermediate-cost modality, offering a balance between accessibility and spatial resolution. Moreover, the possibility of combining fNIRS with techniques such as EEG enhances temporal resolution and provides complementary information about the dynamics of cortical processing [3].

ARHL emerging in adulthood is not merely a peripheral sensory impairment but acts as a potent driver of neural reorganization throughout the auditory network and beyond. Clinical and translational evidence indicates that ARHL is associated with accelerated cognitive decline and increased dementia risk, supporting its role as a central condition rather than a purely sensory deficit [4,5]. Crossmodal plasticity, defined as the recruitment of traditionally auditory cortical regions by visual or somatosensory modalities, is well established in both congenital and acquired deafness [6,7]. However, plasticity arising during early development reflects large-scale cortical activation patterns within sensitive periods, whereas plastic changes in adulthood are constrained by a mature and stabilized neural system. In adults, crossmodal recruitment may support compensatory strategies such as enhanced lip-reading and audiovisual (AV) integration [8], but prolonged or excessive visual dominance can compete with auditory processing and is associated with poorer speech outcomes following cochlear implantation [9,10]. The timing of auditory input is crucial: early stimulation during critical developmental windows promotes typical left-hemispheric specialization of speech networks [11,12,13], whereas congenital or prolonged deprivation disrupts lateralization and interhemispheric connectivity [14,15].

In adults with ARHL, degraded auditory input often results in speech-in-noise comprehension difficulties, increases in listening effort [16,17] and recruitment of frontal cortical resources. This pattern has often been interpreted within the framework of the Compensation-Related Utilization of Neural Circuits Hypothesis (CRUNCH), which proposes that aging individuals engage additional neural resources to maintain performance under increasing task demands [18]. However, recent fNIRS and EEG–fNIRS studies challenge a purely compensatory interpretation: bilateral and right-lateralized dorsolateral prefrontal cortex (DLPFC) hyperactivation has been linked to poorer sentence comprehension and reduced efficiency of auditory–motor integration [19]. Conversely, interventional data support the potential for neuroplastic recovery: auditory training in older adults improves speech-in-noise performance while reducing prefrontal overactivation and enhancing temporal–frontal connectivity, demonstrating that fNIRS can objectively monitor rehabilitation-induced plasticity [20,21].

In CI users, fNIRS provides an unprecedented opportunity to study language networks without device-related artifacts. Research shows that adult CI listeners performing phonological awareness and passage comprehension tasks activate classic perisylvian regions in patterns largely comparable to normal hearing (NH) controls, albeit with subtle differences related to device experience and residual hearing [22]. Similarly, during AV speech perception, both CI and NH participants exhibit robust temporal cortex responses, with AV conditions producing characteristic sub-additive activation [23]. Recent pediatric work expands this view: efficient AV integration can be observed in CI children [24], but the balance between visual and auditory inputs may reflect speech and language outcomes [25], while maladaptive over-reliance on visual cues correlates with language delays [26]. Moreover, fNIRS captures stimulus level-dependent responses of the superior temporal gyrus (STG), closely linked to perceived loudness and potentially relevant for CI programming [21,25,26].

Importantly, studies in infants with congenital or unilateral HL show that atypical lateralization and disrupted network efficiency are evident within the first months of life [14,15]. This interpretation is supported by seminal fNIRS work demonstrating that language-related cortical specialization and hemispheric asymmetries can be reliably measured already in the first year of life [27,28]. These early alterations likely shape later auditory outcomes and highlight the urgency of timely intervention. Yet, despite its potential, the current fNIRS literature in HL remains constrained by several important limitations. First, the available evidence is still based on a relatively small number of studies, most of which include modest sample sizes and predominantly cross-sectional or case–control designs. As a result, causal inferences remain limited, and the temporal evolution of cortical changes associated with HL auditory deprivation and rehabilitation cannot yet be adequately characterized.

Second, there is substantial methodological heterogeneity across studies. Existing reports differ markedly in optode placement, anatomical coverage, short-separation correction, preprocessing pipelines, outcome metrics, and experimental paradigms, ranging from speech-in-noise comprehension to AV speech and cognitive screening tasks. This variability reduces comparability across studies and currently precludes robust quantitative synthesis or meta-analytic integration.

Third, interpretation of fNIRS findings in adult and older populations is further complicated by physiological and anatomical confounders. Age-related changes in cerebrovascular reactivity, cortical atrophy, and scalp–cortex distance may influence hemodynamic responses independently of neural activity, while systemic signals such as extracerebral blood flow and cardiovascular fluctuations may contaminate cortical measurements if not adequately controlled. In addition, the limited penetration depth of near-infrared light restricts fNIRS to superficial cortical regions, preventing direct assessment of subcortical auditory structures and deeper network nodes involved in auditory and cognitive integration.

Finally, although developmental and pediatric studies provide an important interpretative approach for auditory plasticity, the evidence specifically addressing cognition in children with HL remains limited and did not meet the eligibility criteria of the present review. Taken together, these limitations indicate that current conclusions should be considered preliminary and reinforce the need for larger, longitudinal, methodologically standardized studies capable of linking cortical signatures to clinically meaningful auditory and cognitive outcomes.

Given these strengths and gaps, this article aims to provide a structured and comparative review of fNIRS findings in ARHL and CI populations, critically apprising evidence on cortical plasticity, speech perception, and listening effort in adults with ARHL and CI users. It explicitly avoids proposing a unified mechanistic framework and acknowledges relevant background from developmental and crossmodal research without implying mechanistic continuity. ARHL and CI users represent distinct and non-equivalent neurobiological conditions, which are examined separately in this review. These conditions differ in developmental timing, plasticity constraints, and responses of cortical activation patterns. Therefore, similarities in cortical activation patterns should not be interpreted as evidence of shared underlying mechanisms. Understanding whether frontal overactivation represents inefficiency or true compensation, how training reshapes listening effort, and how cortical responses relate to CI outcomes could inform precision audiology, enabling personalized prognostic counseling, optimized rehabilitation, and early risk stratification.

The level of inference is therefore limited to descriptive and methodological observations rather than cross-population mechanistic conclusions.

Our research question, therefore, is to describe population-specific patterns of cortical reorganization and to explore their clinical translation through the use of fNIRS in hearing-impaired patients.

## 2. Materials and Methods

The systematic review was conducted according to the PRISMA 2020 checklist [29]. The study protocol has been stored in the Open Science Framework (OSF) and is available under the DOI https://doi.org/10.17605/OSF.IO/28HYC.

The literature review was conducted through the following steps: identification of the research questions through the PIOS (Population, Intervention, Outcome, Study design) method, literature search, paper selection, appraisal of findings, and summary building.

Eligible studies needed to meet the following criteria: (1) population: hearing-impaired patients; (2) intervention: inclusion of studies that employed fNIRS to investigate auditory and/or cognitive processing (e.g., speech perception, listening effort, neuroplasticity, crossmodal reorganization); (3) outcome: to describe how fNIRS characterizes cortical activity associated with HL including compensatory or maladaptive reorganization, cognitive effort during listening, and potential biomarkers predictive of auditory rehabilitation outcomes; (4) design: randomized controlled trials (RCTs), cohort studies or case–control studies.

Only peer-reviewed articles in English were considered. Theses, posters, commentaries, meta-analyses, letters to the editor, reviews, books, conference papers, study protocols, technical reports and case reports were excluded.

To identify clinical studies on fNIRS findings, the manuscripts were searched on PubMed Central (National Center for Biotechnology Information, Bethesda, MD, USA), Web of Science, and Scopus. The search strings used and the number of articles found are reported in Table 1.

The literature search was conducted in August 2025. No temporal restrictions were applied in order to ensure a comprehensive retrieval of the available evidence, encompassing both early foundational studies and more recent contributions relevant to the topic.

Two independent researchers with more than 10 years of experience in the specific field of the research (G.F., V.D.V.) screened titles and abstracts according to the search strategy focused on the relationship among [(functional near-infrared spectroscopy)] and [(cognition)] and [(hearing)]. First, the researchers read the titles and abstracts of the articles and selected those that were interesting to be as inclusive as possible. Following the first phase, they independently assessed the full text of all potentially relevant studies for inclusion in this review. Any disagreement was resolved through discussion with a third author (biostatistician, N.S.). Then, using a standardized data collection form, the following information was extracted from the included studies: 1st author, journal, publication year, title, database, study aim, type of study and sample size, and results.

The inclusion criteria were primary research studies (including descriptive and observational studies, RCTs, and basic science articles) published on the use of fNIRS for assessing the relation between hearing pathology and cognition. We excluded articles that did not meet the predefined inclusion criteria. Many studies identified in broader searches (e.g., Google Scholar) were excluded because they included only NH participants, lacked an explicit auditory and cognitive focus, or did not report sufficiently characterized HL or did not focus on the use of NIRS. In addition, excluded articles were classified by primary reason for exclusion and by article type (e.g., reviews, conference papers, case reports, and methodological studies), and their counts were recorded to improve transparency of the screening process. The classification of exclusion reasons was performed independently by the reviewers and discrepancies were resolved by consensus.

The Effective Public Health Practice Project Quality Assessment Tool (EPHPP) was developed for systematic reviews of public health topics and can be used in different study designs. It is characterized by six components: “selection bias”, “study design”, “confounding factors”, “blinding”, “data collection methods”, and “withdrawals and dropouts”. The partial and global scores assigned for the assessment of quality level were weak, moderate, or strong [30].

## 3. Results

**Figure 1:**
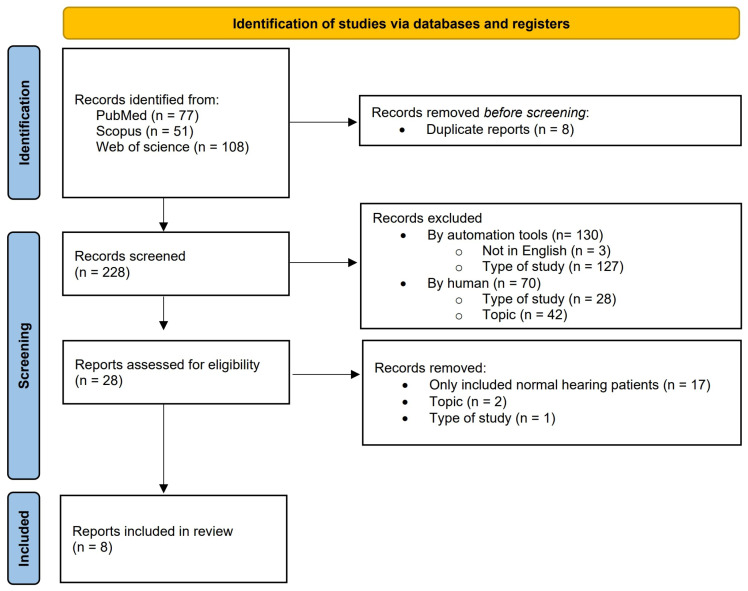
PRISMA flow diagram 2020. The flowchart displays article search and selection [29].

Overall, our search yielded 236 records across all databases. After removal of duplicate records (*n* = 8), 228 studies were screened based on title and abstract. At this stage, 200 records were excluded, including 130 by automation tools and 70 by manual screening. In particular, 155 articles were excluded due to the study design (35 narrative reviews and 2 systematic reviews, 23 conference papers, 2 book chapter, 1 case report, 1 study protocol, 21 preclinical studies, and 70 others, i.e., all other types of study described in PubMed), and 3 were in other languages (1 Spanish, 1 Korean, 1 Turkish). The remaining 28 reports underwent full-text assessment for eligibility. Of these, 20 studies were excluded for the following reasons: inclusion of only normal-hearing participants (*n* = 17), lack of relevance to the topic (*n* = 2), and study design not meeting eligibility criteria (*n* = 1). Ultimately, eight studies met all inclusion criteria and were included in the qualitative review, with no attempt to integrate findings across populations into a unified mechanistic framework. The study selection process is summarized in the PRISMA flow diagram (Figure 1), and a detailed list of studies excluded at the final screening stage is reported in Appendix A. The relatively small number of included studies reflects the strict application of predefined eligibility criteria rather than limited literature availability.

In Table 2 we report the description of all included papers, describing first author, title, journal, publication year, database, aim of the study, type of study, sample size, tools used, stimuli, task details, methodological profile and study results.

### The Quality Assessment Results

Two researchers (R1: V.D.V. and R2: G.F.) independently conducted the quality assessment of the articles included in our study. Both reviewers were researchers with expertise in reviewing methods and critical appraisal, using the EPHPP [30]. Each reviewer independently assigned a quality rating to each study. In cases of disagreement between R1 and R2, ratings were discussed and subsequently adjudicated by a third reviewer (N.S.), a biostatistician with more than 20 years of experience, who provided the final quality assessment. Any discrepancies in title/abstract sorting were resolved by majority decision. The quality ratings of all included studies, including individual reviewer scores and final assessments, are reported in Table 3.

## 4. Discussion

The studies included in this systematic review consistently show that fNIRS is a reliable method for exploring auditory and cognitive cortical dynamics in adults with HL. To move beyond a purely descriptive synthesis, the findings of this review are interpreted within a structured analytical approach based on three key dimensions: (i) cortical targeting and optode placement, (ii) experimental paradigms and stimulus characteristics, and (iii) participant-related factors influencing the fNIRS signal. These dimensions are explicitly summarized in Table 2, which enables direct comparison of methodological variability across studies. What emerges is that the functional significance of the observed activations is not uniform and changes according to the clinical and experimental design. Importantly, prefrontal activation patterns should not be interpreted as uniform across tasks or populations. Rather, their functional meaning appears to be strongly task-dependent and modulated by both cognitive demand and clinical characteristics, including degree of HL, age, and listening context.

In ARHL, several investigations reported increased activation of the DLPFC during speech-in-noise listening [16,19]. Traditionally interpreted as a sign of compensatory recruitment, this pattern now appears more closely linked to poorer comprehension and greater listening effort, supporting the view that it reflects maladaptive neural reorganization rather than successful compensation.

Although several studies included in this review generically refer to “prefrontal cortex” (PFC) activation, a closer inspection of optode placement and channel-level analyses reveals that, when signal quality and spatial sampling allow, fNIRS findings consistently converge on specific prefrontal subregions rather than on an undifferentiated frontal response. In particular, the DLPFC emerges as the most frequently and robustly implicated area during effortful speech perception and speech-in-noise processing in adults with ARHL [19,20,21].

Studies employing high-density or anatomically guided montages explicitly localized increased task-related hemodynamic responses to bilateral or right-lateralized DLPFC, and linked this activation to poorer behavioral performance and greater listening effort, supporting the interpretation of frontal recruitment as neural inefficiency rather than successful compensation [19]. Conversely, investigations combining fNIRS with electrophysiological measures further refined this pattern by demonstrating a decompensation of left DLPFC activity and weakened frontoparietal connectivity in more advanced stages of HL, suggesting a progressive breakdown of top-down auditory–motor control mechanisms [20].

Beyond the DLPFC, other prefrontal subregions have been reported depending on task demands and cognitive load. Studies focusing on executive control and cognitive screening in hearing-impaired older adults described additional involvement of ventrolateral, frontopolar, and orbitofrontal cortices, likely reflecting the engagement of domain-general executive and monitoring processes [22]. However, the reliability of such fine-grained anatomical distinctions remains constrained by intrinsic limitations of fNIRS, including variable optode configurations, depth sensitivity, and signal-to-noise ratio (SNR), which do not always permit consistent separation of adjacent prefrontal areas across studies [21,22].

Taken together, available evidence indicates that prefrontal activation predominantly reflects DLPFC involvement, with additional regions recruited as task demands increase. Explicit reporting of optode localization and standardized prefrontal parcellation will be essential in future studies to further clarify the functional role of distinct prefrontal subregions in listening effort and auditory and cognitive plasticity [19,20,21,22].

Although several studies reported statistically significant associations, the interpretation of effect sizes requires careful consideration of their methodological robustness. As shown by the EPHPP assessment (Table 3), the overall quality of the included studies was predominantly moderate to weak, with recurrent limitations in selection bias, control of confounders, and blinding. For this reason, reported effects were interpreted using a quality-weighted narrative approach, giving greater weight to findings derived from studies with stronger ratings in study design and data collection, while results from weaker studies were considered mainly in terms of direction and consistency rather than magnitude. In addition, a formal meta-analysis was not undertaken because substantial clinical and methodological heterogeneity—including differences in outcome definitions, measurement instruments, and statistical metrics—would have rendered any pooled effect estimate potentially misleading rather than informative.

In moderate to severe ARHL, reduced left DLPFC activity and weakened frontoparietal connectivity have also been described [20,31,32,33], indicating that the top-down auditory–motor control network may progressively decompensate as sensory decline advances. Importantly, longitudinal evidence shows that targeted auditory training can modify these cortical dynamics: prefrontal overactivation diminishes and temporal–frontal coupling strengthens in parallel with improved speech-in-noise performance [20,21]. A further conceptual issue that warrants consideration concerns the origin of the inefficient DLPFC activation patterns observed in adults with HL. Specifically, it remains unclear whether these frontal alterations primarily reflect a state-dependent response to ongoing auditory degradation or whether they also represent a long-term neural signature shaped by earlier periods of reduced or abnormal auditory input.

Although pediatric and developmental studies were not included in the present review, evidence from these populations provides important mechanistic insights into long-term auditory–cognitive plasticity.

Evidence from developmental studies suggests that auditory deprivation during sensitive periods can induce persistent changes in cortical organization, including altered hemispheric specialization and large-scale network efficiency. These developmental findings are particularly relevant in the context of cross-modal plasticity, where sensory deprivation leads to the recruitment of non-auditory cortical resources, a mechanism that may persist into adulthood. In this context, the increased reliance on prefrontal resources documented in adult fNIRS studies may not be solely attributable to current listening demands, but may instead reflect the cumulative impact of long-standing adaptations within auditory and cognitive systems.

Importantly, the predominantly cross-sectional nature of the available literature precludes a clear disentanglement of these patterns. As such, the interpretation of DLPFC hyperactivation as either compensatory or inefficient should be considered within a broader developmental perspective. Longitudinal cohort studies, ideally following individuals with HL across different stages of life, will be crucial to clarify the temporal dynamics of these cortical changes and to determine whether adult fNIRS signatures reflect transient adaptation, progressive neural reorganization, or a developmental imprint of early auditory experience. Such findings reinforce the potential of fNIRS as an objective biomarker to monitor neuroplastic benefits of rehabilitation.

A central contribution of fNIRS research in HL lies in its ability to jointly examine auditory and cognitive components within specific experimental contexts. Speech understanding in adverse listening conditions places substantial demands on executive functions, working memory, and attentional control, particularly in older adults with degraded auditory input [16,17]. fNIRS studies consistently show that increased recruitment of prefrontal regions during speech perception reflects not only sensory compensation but also the engagement of domain-general cognitive control networks.

Indeed, recent evidence indicates that prefrontal overactivation may serve as a neural marker of cognitive vulnerability rather than successful compensation [34]. Studies combining fNIRS with cognitive screening tools have demonstrated that reduced frontal hemodynamic responses during speech tasks are associated with poorer cognitive status and increased risk of mild cognitive impairment in hearing-impaired older adults [22,31,32,33]. These findings support the notion that fNIRS-derived measures of listening effort may provide preliminary markers of cognitive vulnerability, although current evidence remains limited and does not yet support predictive clinical use beyond what is captured by audiometric thresholds alone. Further longitudinal and large-scale studies are required to determine whether fNIRS-derived measures can reliably predict cognitive decline or should instead be interpreted as correlates of increased listening effort and reduced cognitive reserve. From this perspective, fNIRS aligns well with contemporary models linking HL, increased cognitive load, and dementia risk. Evidence suggests that HL may contribute to cognitive deterioration through both increased listening-related cognitive burden and shared neurobiological patterns, including oxidative stress, vascular dysfunction, and neurodegenerative processes [4,5,35].

By offering objective, task-based measures reflecting the interaction between auditory demands and cognitive load within specific paradigms, fNIRS may help disentangle peripheral audibility, inefficient cortical compensation, or reduced cognitive reserve. This has direct clinical implications, as it suggests a potential role for fNIRS in identifying individuals who may benefit from combined auditory and cognitive rehabilitation strategies, rather than hearing-focused interventions alone [17,22].

In CI users, cortical activation patterns reflect a different neurobiological context. Adult CI listeners performing phonological awareness and language comprehension tasks generally show activity patterns similar to those of NH peers, though with slightly reduced accuracy and modulation by device experience [22,26]. AV speech perception engages the typical temporo-frontal language network without major abnormalities [1,24,25], confirming the applicability of fNIRS even in the presence of implants. Importantly, ARHL and CI users represent distinct neurobiological conditions and should not be considered interchangeable models of HL. Importantly, they should also not be assumed to share common patterns of cortical plasticity. Any apparent similarity in fNIRS activation patterns within these populations may reflect different underlying processes rather than a shared computational consequence of HL and must be interpreted cautiously. ARHL reflects a gradual, late-onset sensory degradation within a mature neural system, whereas cochlear implantation involves partial restoration of auditory input following a period of sensory deprivation, often with different developmental trajectories and plasticity responses. In this review, these populations are therefore discussed separately, and their comparison is intended to highlight both apparently similar yet mechanistically distinct patterns of auditory and cognitive adaptation to degraded input.

Developmental fNIRS evidence can provide external contextual insights, although it should not be interpreted as mechanistically continuous or directly comparable to adult cortical responses with adult findings in cochlear implanted children. Although these studies fall outside the scope of the present review, they provide valuable context for interpreting long-term plasticity responses.

A useful structured analytical approach for these adult findings comes from developmental fNIRS evidence in cochlear-implanted children. A recent review shows that fNIRS can reliably capture auditory, AV, and cross-modal cortical responses in pediatric CI users, providing mechanistic insights into sensitive periods, auditory deprivation, and reorganization of temporo-frontal networks [36]. Importantly, developmental patterns of cross-modal recruitment and network efficiency are linked to later auditory–language outcomes, supporting the view that the frontal engagement and listening-related cognitive load observed in adults may reflect long-term plastic adaptations to degraded input, rather than a purely short-term compensatory response [36].

Insights from pediatric research add an important developmental perspective: visual recruitment of auditory regions can be beneficial when balanced—supporting AV integration and improved speech outcomes [25]—but becomes maladaptive if excessive, and is then associated with persistent language delays [26]. These results fit well with current understanding of neurodevelopment, where timely auditory input helps maintain left-hemisphere specialization [11,12,13], while congenital deprivation disrupts network efficiency [14,15] and can restrict the capacity for later CI-driven reorganization [6].

Another clinically relevant observation comes from the study by Sheffield et al. [24], showing that STG activation is strongly modulated by stimulus level. Accounting for loudness perception is therefore critical when interpreting fNIRS findings and may also guide CI fitting and gain adjustment. In combination with AV paradigms [24], this line of research points toward the future development of future clinically oriented research, where device programming and auditory training could be tailored to individual cortical response profiles. In this context, it is also relevant to consider how fNIRS findings relate to established electrophysiological measures used in CI candidacy and follow-up, such as electrically evoked cortical auditory evoked potentials (eCAEPs) and electrically evoked auditory late responses (EALRs). While these techniques provide objective information on the presence and timing of cortical responses to electrical stimulation, fNIRS offers complementary insight into the spatial organization and functional recruitment of cortical networks during more ecologically valid tasks, including speech-in-noise perception and AV speech processing [1,22,25,26]. Therefore, electrophysiological measures may be particularly useful to verify effective cortical activation, whereas fNIRS may contribute to understanding how efficiently auditory input is processed within broader auditory and cognitive systems. In a multimodal perspective, the integration of these approaches could support more comprehensive CI assessment and optimization, particularly in cases where behavioral measures are limited or inconclusive.

Preoperative cortical markers identified by fNIRS—such as visual takeover of the STG or indices of frontal listening effort—are also emerging as tools to refine prognostic models beyond standard demographic predictors [2,10]. This approach could support biologically informed counseling and more individualized rehabilitation planning, helping clinicians identify patients who might benefit from early intervention or intensified auditory training.

Nonetheless, the field still faces important methodological challenges that must be considered when interpreting fNIRS findings in adults with HL. A formal quantitative synthesis was not undertaken because the included studies showed substantial clinical and methodological heterogeneity, including differences in participant characteristics, outcome definitions, experimental paradigms, optode placement, and statistical reporting, which would have rendered any pooled effect estimate potentially misleading rather than informative.

This structured narrative synthesis therefore reflects a necessary methodological choice given the current state of the evidence, rather than a limitation of the review analysis. Within this perspective, variability across studies can be descriptively organized along three main dimensions: (i) cortical regions of interest and optode placement, (ii) experimental paradigms and stimulus characteristics, and (iii) participant-related factors influencing the hemodynamic response.

Importantly, participant-related and physiological factors represent a critical but often underreported source of variability in fNIRS studies. Age-related vascular changes, cortical atrophy, and increased scalp–cortex distance, together with individual differences in neurovascular coupling, can significantly affect signal interpretation, particularly in older adults with HL. These factors complicate the interpretation of cortical activation patterns, as hemodynamic responses may not directly reflect underlying neural efficiency. Moreover, differences in hearing status (e.g., ARHL severity, CI experience, and duration of auditory deprivation) further contribute to between-study variability.

In addition, methodological heterogeneity across studies—including differences in optode placement, signal processing pipelines, and experimental paradigms—limits quantitative comparability and the derivation of robust normative benchmarks. Systemic physiological signals, such as scalp blood flow and cardiovascular fluctuations, may further contaminate fNIRS measurements, particularly during cognitively demanding speech-in-noise tasks. Greater standardization of acquisition and analysis protocols is therefore required.

These methodological sources of variability are compounded by broader reporting limitations that may further constrain interpretation and external validity. In fNIRS research on speech and language-related clinical populations, systematic evidence indicates frequent underreporting of sociodemographic variables, language background, and participant exclusion/attrition reasons—factors that directly affect reproducibility and generalizability. This is particularly relevant for hearing- and communication-related disorders, where linguistic experience and developmental trajectories can meaningfully shape cortical responses during speech processing and effortful listening [37].

Moreover, the intrinsic limitation of near-infrared light penetration restricts reliable measurements to superficial cortical regions, preventing direct assessment of subcortical auditory structures and deeper network nodes involved in auditory and cognitive integration.

For these reasons, multimodal approaches combining fNIRS with complementary techniques such as EEG [3] or fMRI may help improve spatial and temporal resolution and strengthen the validation of fNIRS-derived biomarkers. Addressing these methodological challenges will be essential for moving fNIRS from an exploratory research tool into a reliable clinical instrument to assess auditory processing, listening effort, and cognitive vulnerability in adult populations.

In this context, interpretation of fNIRS findings remains constrained by methodological heterogeneity, including variability in experimental paradigms, participant characteristics, and cortical coverage. As a result, it is still unclear to what extent observed activation patterns reflect auditory processing, cognitive load, or more general acoustic factors. Future research should aim to standardize task designs and acquisition protocols, as well as to adopt longitudinal and clinically oriented approaches, in order to clarify the functional significance of fNIRS measures and support their potential translation into clinical practice.

Finally, the level of inference supported by the present synthesis is primarily descriptive and methodological. Given the heterogeneity of populations, experimental paradigms, and outcome measures, the current evidence does not allow for mechanistic generalizations or cross-population equivalence claims. Rather, the findings should be interpreted as context-dependent observations of cortical activation patterns under specific experimental conditions.

### Limitations

This review has some limitations that should be acknowledged. First, the number of studies meeting the inclusion criteria was relatively limited (*n* = 8). This reflects both the specificity of the review focus and the still developing nature of fNIRS applications in hearing-impaired populations. While the search strategy was comprehensive, the restricted evidence base may limit the generalizability of the findings and does not allow for quantitative synthesis. Although this review deliberately focused on adult populations, an important gap in the literature concerns pediatric HL. Early cortical development and sensitive periods are known to play a decisive role in auditory plasticity and rehabilitation outcomes [6,12,14]. While pediatric studies on HL do exist, fNIRS evidence specifically addressing cognitive outcomes remains limited and did not meet the eligibility criteria of the present review. They are discussed here only to provide a developmental perspective on auditory and cognitive plasticity. Future research should therefore extend the application of fNIRS to infants and children with HL to better characterize developmental trajectories and validate neural biomarkers that could inform early intervention strategies.

Beyond age-related considerations, the available fNIRS literature is characterized by substantial methodological heterogeneity, including differences in optode montages, signal processing pipelines, and task paradigms. More specifically, heterogeneity emerged across three main dimensions. First, experimental paradigms varied substantially, ranging from speech-in-noise perception and AV integration tasks to language processing and cognitive paradigms, each engaging partially distinct neural systems and levels of cognitive demand. Second, the included populations were not fully homogeneous, encompassing individuals with different degrees of HL age ranges and clinical profiles (e.g., ARHL versus CI users), which are associated with distinct patterns of functional cortical responses and cognitive involvement. Third, considerable variability was observed in fNIRS acquisition protocols, including differences in optode configurations, spatial coverage (e.g., temporal versus frontotemporal montages), and preprocessing pipelines, all of which may influence signal quality, anatomical specificity, and the comparability of reported activation patterns. This variability complicates direct comparisons across studies and precludes robust quantitative synthesis or meta-analytic approaches. Moreover, most existing evidence is cross-sectional, underscoring the need for longitudinal studies capable of tracking neuroplastic changes over time and directly linking cortical signatures to functional hearing and speech outcomes.

This structured synthesis provides a level of cross-study interpretation that cannot be obtained from individual study reports alone, particularly in identifying consistent methodological patterns and sources of variability across the field.

An additional limitation concerns the conceptual heterogeneity of the included populations. ARHL and cochlear implantation involve fundamentally different neurobiological conditions, and their inclusion within the same review does not imply mechanistic continuity. This review therefore does not aim to provide an integrated model of auditory and cognitive plasticity, but rather to document how fNIRS findings vary within distinct clinical contexts. This conceptual heterogeneity reflects a fundamental characteristic of the field and constrains the level of inference of the present review.

## 5. Conclusions

This systematic review highlights the value of fNIRS as a robust and versatile tool for investigating cortical responses associated with HL in specific populations. In ARHL, fNIRS consistently reveals altered frontotemporal dynamics and increased reliance on prefrontal resources, supporting the interpretation of frontal recruitment as a marker of listening effort and maladaptive neural reorganization rather than effective compensation. In CI users, fNIRS demonstrates preserved activation of language-related cortical networks while remaining sensitive to stimulus level and AV integration, confirming its applicability in populations traditionally excluded from conventional neuroimaging.

Importantly, longitudinal evidence indicates that fNIRS can track experience- and training-related neuroplastic changes, with reductions in prefrontal overactivation and strengthening of temporal–frontal coupling paralleling improvements in speech-in-noise performance. Beyond auditory processing, fNIRS provides insight into the interaction between HL and cognition by offering task-based measures of listening effort and executive resource engagement. In this context, fNIRS-derived cortical markers may help identify individuals at increased risk of cognitive vulnerability and support more targeted rehabilitation strategies.

Therefore, the conclusions of this review should be interpreted with caution, as they are limited by the methodological heterogeneity and overall low quality of the available evidence. Large-sample, prospective, multicenter studies—ideally randomized controlled designs—are needed to strengthen and validate these findings. Such efforts will be essential to capture developmental trajectories of auditory cortical responses and to translate fNIRS into a clinically meaningful tool for precision audiology in specific hearing-impaired populations. Importantly, current evidence does not support unified mechanistic interpretations across different forms of HL, and therefore the review should not be interpreted as evidence that these conditions can be meaningfully integrated within a single auditory and cognitive framework. Future work should avoid assuming continuity across different forms of HL and instead adopt population-specific frameworks.

## Figures and Tables

**Table 1:** Database, search strings and number of articles found.

Database	Search Strings	Number of Results
PubMed	(((((“functional”[All Fields] OR “functional s”[All Fields] OR “functionalities”[All Fields] OR “functionality”[All Fields] OR “functionalization”[All Fields] OR “functionalizations”[All Fields] OR “functionalize”[All Fields] OR “functionalized”[All Fields] OR “functionalizes”[All Fields] OR “functionalizing”[All Fields] OR “functionally”[All Fields] OR “functionals”[All Fields] OR “functioned”[All Fields] OR “functioning”[All Fields] OR “functionings”[All Fields] OR “functions”[All Fields] OR “physiology”[MeSH Subheading] OR “physiology”[All Fields] OR “function”[All Fields] OR “physiology”[MeSH Terms]) AND (“spectroscopy, near infrared”[MeSH Terms] OR (“spectroscopy”[All Fields] AND “near infrared”[All Fields]) OR “near-infrared spectroscopy”[All Fields] OR (“near”[All Fields] AND “infrared”[All Fields] AND “spectroscopy”[All Fields]) OR “near infrared spectroscopy”[All Fields])) OR “fNIRS”[All Fields]) AND (“spectroscopy, near infrared”[MeSH Terms] OR (“spectroscopy”[All Fields] AND “near infrared”[All Fields]) OR “near-infrared spectroscopy”[All Fields] OR (“near”[All Fields] AND “infrared”[All Fields] AND “spectroscopy”[All Fields]) OR “near infrared spectroscopy”[All Fields])) OR (“spectroscopy, near infrared”[MeSH Terms] OR (“spectroscopy”[All Fields] AND “near infrared”[All Fields]) OR “near-infrared spectroscopy”[All Fields] OR “nirs”[All Fields])) AND (“cognition”[MeSH Terms] OR “cognition”[All Fields] OR “cognitions”[All Fields] OR “cognitive”[All Fields] OR “cognitively”[All Fields] OR “cognitives”[All Fields]) AND (“hearing”[MeSH Terms] OR “hearing”[All Fields] OR “hearings”[All Fields])	77
Scopus	(TITLE-ABS-KEY(nirs) OR TITLE-ABS-KEY(near infrared spectroscopy) OR TITLE-ABS-KEY(fnirs) OR TITLE-ABS-KEY(fnirs functional near infrared spectroscopy) AND TITLE-ABS-KEY(cognition) AND TITLE-ABS-KEY(hearing))	51
Web of Science	(NIRS OR fNIRS OR near-infrared spectroscopy OR functional near-infrared spectroscopy) AND cognition AND hearing	108

**Table 2:** Description of included papers.

First Author, Title, Journal (Year)	Database	Topic Aim of the Study	Type of Study, Sample Size, Tools	Stimuli, Task Details	Methodological Profile	Results
Perron et al. Age-related increased frontal activation in sentence comprehension reflects inefficiency, not compensation *Neurobiology of aging* (2025) [19]	Scopus	To investigate whether age-related increases in PFC activation during sentence comprehension in noise reflect compensatory neural mechanisms or inefficient processing.	Observational cross-sectional study. 57 adults (22 younger, 35 older; mean age of older group 73.9 ± 4.8 years). Pure-tone audiometry, MoCA, fNIRS	Adapted Revised Speech-in-Noise task; sentences with high vs. low predictability; 2 SNR levels (+4, −2 dB); babble noise; active word report	PFC montage; speech-in-noise task; older adults	Older adults exhibited increased bilateral, predominantly right-lateralized, PFC activation as SNR decreased. Higher PFC activation was associated with poorer task accuracy. Mediation analyses indicated that prefrontal overactivation partially accounted for age-related performance decline, with hearing thresholds and cognitive status modulating the relationship between SNR and cortical activation.
Wang et al. Impact of age-related hearing loss on decompensation of left DLPFC during speech perception in noise: a combined EEG-fNIRS study *GeroScience* (2025) [20]	Scopus	To examine the effects of ARHL on top-down auditory–motor integration and frontal cortical control during speech perception in noise.	Case–control study. 78 older adults: 26 NH (65.4 ± 2.8 years), 26 mild HL (66.3 ± 3.8 years), 26 moderate–severe HL (67.5 ± 3.7 years). Pure-tone audiometry, MoCA, MMSE, Geriatric Depression Scale, UCLA Loneliness Scale; synchronous EEG–fNIRS	Mandarin hearing in noise Test; quiet and 2 SNR levels (0 and +5 dB); speech-shaped noise; active speech perception and passive listening blocks	Left DLPFC-focused montage; speech-in-noise task; older adults with ARHL	Participants with ARHL showed reduced left DLPFC activation and attenuated theta-band oscillatory activity under noisy conditions compared with NH controls. Decreased frontoparietal connectivity correlated with high-frequency HL severity and poorer speech-in-noise performance, supporting a model of decompensation of top-down auditory–motor control with increasing sensory decline.
Lee at al. Functional near-infrared spectroscopy analysis of the cognitive functions of elderly patients with hearing loss *Journal of audiology and otology* (2025) [22]	Scopus	To compare frontal cortical activation patterns in older adults with moderate–severe bilateral HL according to cognitive status, focusing on the ability of fNIRS to discriminate individuals at risk for MCI.	Prospective observational study. 100 older adults (>50 years) with bilateral moderate–severe SNHL; subdivided into normal cognition and MCI-risk groups based on Clinical Dementia Rating and Geriatric Depression Scale. Pure-tone audiometry, MoCA, MMSE, Geriatric Depression Scale, UCLA Loneliness Scale; synchronous EEG–fNIRS	Cognitive tasks including verbal fluency, picture naming, working memory (auditory word recall), delayed recall, and Stroop task; auditory word stimuli (60–65 dB SPL) for memory task; active responses; block design with rest intervals	Prefrontal (DLPFC/VLPFC/OFC) montage; cognitive task battery; older adults with ARHL	Participants with preserved cognition demonstrated significantly higher oxygenated hemoglobin responses in bilateral DLPFC, VLPFC, FPC, and OFC across most tasks compared with the MCI-risk group. fNIRS measures effectively differentiated cognitive status in individuals with HL.
Kovelman et al. Words in the bilingual brain: an fNIRS brain imaging investigation of lexical processing in sign-speech bimodal bilinguals *Frontiers in human neuroscience* (2014) [23]	Scopus	To assess how bimodal bilingual experience (sign language and spoken language) influences the neural organization of lexical processing.	Case–control study. 29 adults: 5 hearing sign language signers–English bimodal bilinguals, 7 deaf sign language signers, 17 English monolinguals Pure-tone audiometry, speech audiometry, fNIRS	Linguistic stimuli consisting real and pseudo-signs in sign language; visual language processing task; passive observation	Left frontotemporal language network; sign language processing task; bimodal bilingual and Deaf participants	Bimodal bilinguals showed reduced left parietal activation compared with deaf signers during exclusive sign language use, but increased left temporo-parietal activation relative to monolinguals during language switching tasks. These findings indicate experience-dependent plasticity of temporo-parietal language networks associated with bimodal bilingualism.
Mai et al. Neuroplasticity of speech-in-noise processing in older adults assessed by functional near-infrared spectroscopy (fNIRS) *Brain topography* (2024) [21]	WOS	To investigate neuroplastic changes in speech-in-noise processing in older adults following auditory training, using fNIRS as a neural outcome measure.	Interventional longitudinal study. 10 older adults (63–78 years, mean age 70 years; with mostly mild–moderate HL) Behavioral assessment, speech-in-noise training; fNIRS	Speech in noise; adaptive listening task Adaptive sentence lists (ASL) in 8-talker babble; adaptive SNR (SRT); active word report; speech vs. spectrally rotated stimuli; block design	Bilateral frontotemporal montage; speech-in-noise and auditory processing tasks; older adults with ARHL	Auditory training led to improved speech-in-noise performance, particularly evident at 4-week retention. Neuroimaging revealed reduced left frontal and prefrontal activation suggesting decreased listening effort, suppression of noise-related auditory cortex responses, and increased functional connectivity across temporal–parietal–frontal networks, demonstrating training-induced neuroplasticity detectable by fNIRS.
Sheffield et al. Sound level changes the auditory cortical activation detected with functional near-infrared spectroscopy *Brain topography* (2023) [24]	WOS	To determine the influence of sound level on auditory cortical activation measured with fNIRS in NH individuals and CI users.	Case–control study. 29 adults: 16 NH (40–64 years, mean age 50.1 years) and 13 bilateral CI users (23–67 y, mean 49.1) fNIRS	Signal-correlated noise (speech-like stimuli derived from AzBio sentences); 4 intensity levels (45, 55, 65, 75 dBA) plus silence; event-related passive listening (fNIRS) and loudness rating task	Left frontotemporal (auditory cortex) montage; auditory stimulation (intensity modulation); NH and CI participants	Left STG activation increased with stimulus intensity in both groups. In CI users, cortical activation was also significantly correlated with perceived loudness. These results underscore the importance of controlling for sound level and subjective loudness in fNIRS studies of auditory processing.
van de Rijt et al. Temporal cortex activation to audiovisual speech in normal-hearing and cochlear implant users measured with functional near-infrared spectroscopy *Frontiers in human neuroscience* (2016) [25]	WOS	Temporal Cortex Activation to AV Speech in Normal-Hearing and CI Users Measured with fNIRS	Observational study. 33 NH adults (18–62 years, median age 29 years) and 5 post-lingually deaf unilateral CI users (55–59 years, all female). fNIRS	AV speech stimuli (Dutch story segments); auditory-only, visual-only, and AV conditions; passive listening/viewing with comprehension check	Temporal cortex (STG) coverage; AV speech task; NH and CI participants	Both NH and CI users exhibited significant temporal cortex activation for auditory, visual, and AV speech, with stronger responses for auditory compared with visual stimuli. AV responses showed sub-additive effects, indicating saturation. The study confirmed the feasibility of fNIRS for cortical speech processing assessment in CI users.
Bisconti et al. Functional near-infrared spectroscopy brain imaging investigation of phonological awareness and passage comprehension abilities in adult recipients of cochlear implants *Journal of speech, language, and hearing research* (2016) [26]	WOS	To investigate cortical responses during phonological awareness and passage comprehension in adult CI users compared to NH controls.	Case–control study. 10 adult CI users (mean age 52.7 ± 17.3 years) vs. 10 NH (mean 50.6 ± 17.2 years); right-handed and without neurological disease. language and reading assessments, fNIRS	Language tasks including phonological awareness and passage comprehension; visually presented words and text; active responses	Frontotemporal montage; language tasks; CI users	Both groups demonstrated largely overlapping activation patterns across classical language-related cortical regions. CI users showed slightly reduced accuracy but comparable reaction times relative to controls. Findings suggest preserved large-scale language networks in adult CI users and support the feasibility of fNIRS for studying higher-order language processing in this population.

Legend: ARHL, age-related hearing loss; CI, cochlear implant; DLPFC, dorsolateral prefrontal cortex; EEG, electroencephalography; fNIRS, functional near-infrared spectroscopy; HL, hearing loss; MMSE, Mini-Mental State Examination; MoCA, Montreal Cognitive Assessment; NH, normal hearing; OFC, orbitofrontal cortex; PFC, prefrontal cortex; SNR, signal-to-noise ratio; STG, superior temporal gyrus; VLPFC, ventrolateral prefrontal cortex.

**Table 3:** Quality assessment: EPHPP scores.

Author, Year [Ref. Num]	EPHPP Scores												
	SB		D		C		B		DC		DO		Overall
	R1	R2	R1	R2	R1	R2	R1	R2	R1	R2	R1	R2	
Perron et al., 2025 [19]	W	M	W	M	W	M	S	W	M	S	S	M	M
Wang et al., 2025 [20]	M	M	W	M	M	S	M	W	M	S	M	S	M
Lee et al., 2025 [22]	M	M	W	M	W	M	W	W	M	S	W	M	M
Kovelman et al., 2014 [23]	M	W	M	M	W	M	W	W	M	S	M	M	W
Mai et al., 2024 [21]	M	W	M	M	W	M	M	W	M	S	M	M	W
Sheffield et al., 2023 [24]	W	M	W	M	W	M	W	W	W	S	M	M	M
van de Rijt et al., 2016 [25]	W	W	W	M	W	M	W	W	W	S	M	M	W
Bisconti et al., 2016 [26]	W	W	M	M	M	M	W	W	M	S	M	M	W

Legend: SB = Selection Bias, D = Study Design, C = Confounders, B = Blinding, DC = Data Collection Method, DO = Withdrawals and Dropouts; W = Weak, M = Moderate, S = Strong.

## Data Availability

No new data were created or analyzed in this study.

## Abbreviations

The following abbreviations are used in this manuscript:

ARHL	age-related hearing loss
AV	audiovisual
CI	cochlear implant
DLPFC	dorsolateral prefrontal cortex
EEG	electroencephalography
EPHPP	effective public health practice project quality assessment tool
fMRI	functional magnetic resonance imaging
fNIRS	functional near-infrared spectroscopy
FPC	frontopolar cortex
HL	hearing loss
MoCA	Montreal cognitive assessment
NH	normal hearing
OFC	orbitofrontal cortex
PFC	prefrontal cortex
RCTs	randomized controlled trials
SNHL	sensorineural hearing loss
SNR	signal-to-noise ratio
STG	superior temporal gyrus
VLPFC	ventrolateral prefrontal cortex

## Appendix A

### Appendix A.1

Studies including only normal-hearing participants (*n* = 17):

1. https://doi.org/10.1044/2025_JSLHR-24-00839
2. https://doi.org/10.1093/cercor/bhae178
3. https://doi.org/10.1038/s41467-018-04819-z
4. https://doi.org/10.1007/s10162-016-0611-7
5. https://doi.org/10.1016/j.neuroimage.2014.08.034
6. https://doi.org/10.3389/fnins.2014.00373
7. https://doi.org/10.1007/s10162-021-00817-z
8. https://doi.org/10.1016/j.heares.2020.108155
9. https://doi.org/10.1016/j.heares.2018.09.005
10. https://doi.org/10.1016/j.heares.2016.07.007
11. https://doi.org/10.1016/j.heares.2016.01.009
12. https://doi.org/10.1002/hbm.24034
13. https://doi.org/10.1007/s10162-018-0661-0
14. https://doi.org/10.1016/j.neuroimage.2011.01.011
15. https://doi.org/10.1097/AUD.0000000000000836
16. https://doi.org/10.1007/s10548-015-0424-8
17. https://doi.org/10.1016/j.brainres.2025.149561

### Appendix A.2

Studies excluded due to topic irrelevance (*n* = 2):

1. https://doi.org/10.3390/brainsci10070429
2. https://doi.org/10.1016/j.neuron.2010.03.001

### Appendix A.3

Studies excluded due to study design (*n* = 1):

1. https://doi.org/10.3390/audiolres12010001
